# Design and Characterization of a Prototype Pixel Readout Chip for Synchrotron Single Photon-Counting Detectors with 50 µm Pitch and 20 e^−^rms ENC Noise

**DOI:** 10.3390/s26102992

**Published:** 2026-05-09

**Authors:** Shijie Lu, Yifan Jiang, Tao Sun, Fuwan Gan, Tianyang Wang, Zhen Sheng

**Affiliations:** 1 State Key Laboratory of Materials for Integrated Circuits, Shanghai Institute of Microsystem and Information Technology, Chinese Academy of Sciences, 865 Changning Road, Shanghai 200050, China; 2 University of Chinese Academy of Sciences, Beijing 100049, China; 3 Zhangjiang Laboratory, 100 Haike Road, Shanghai 201210, China; 4 School of Information Science and Technology (SIST), ShanghaiTech University, 393 Middle Huaxia Road, Shanghai 201210, China

**Keywords:** X-ray detectors, pixel detectors, ASIC, pixel readout chip, synchrotron radiation sources, low-energy detection

## Abstract

As synchrotron radiation sources (SRSs) expand to cover a broader energy range, the demand for hybrid detectors with improved spatial and energy resolution is increasing. This paper presents the design and characterization of a prototype pixel readout ASIC featuring a small pixel size and low noise, developed for low energy soft X-ray applications. This chip adopts the single photon-counting (SPC) approach and each pixel consists of a front-end amplifier, a discriminator, a charge injection circuitry and a pair of 15-bit counters with associated logic. Fabricated in a 130 nm CMOS process, the chip integrates a 2 × 16 pixel matrix with a 50 µm ×50 µm pixel size. Measurement results indicate the maximum pixel equivalent noise charge (ENC) across the matrix is 20 e^−^rms without sensor attached. The results validate that the chip design has the potential to deliver a low-energy resolution for soft X-ray applications.

## 1. Introduction

Synchrotron radiation sources (SRSs) are capable of generating soft X-rays at ultra-low energies, reaching as low as 150 eV [1]. Low-energy soft X-rays can offer significant advantages in studying specific material structures and provide effective experimental approaches, such as small-angle X-ray scattering experiments [2]. A complete SRS imaging system architecture comprises an X-ray source, a target object, and a hybrid detector. As SRSs evolve to support lower-energy photons, hybrid detectors with resolutions of lower energies become increasingly critical. Hybrid detectors typically consist of a pixel sensor chip and a pixel readout chip that have the same pixel-array geometry and are interconnected by the bump bonding technique. Existing pixel detectors, such as the Medipix-series [3,4] and Eiger-series [5,6], play a key role in the performance of SRS imaging system. These detectors adopt the single photon-counting (SPC) approach and achieve high spatial resolution through small pixel sizes. In the SPC method, each pixel includes an independent front-end electronic readout channel, and the charge signal generated by each impinging photon is processed independently. The advantages of this method include low-noise imaging (appropriately set discrimination threshold cuts background noise) and the ability to count photons only within a given energy window.

The distinguishing feature of the Medipix-series detectors is its charge-summing and discrimination technology: when an incident photon deposits the charge across multiple pixels, an arbitration network identifies which pixel received the largest charge, and the charges shared by neighboring pixels are summed and reconstructed onto that pixel [3,4]. The Eiger-series detectors employ instant retrigger technology to mitigate photon pile-up: when multiple photons arrive simultaneously the resulting digital pulse overlaps and broadens, which can produce a single count instead of multiple counts, and thereby paralyze the counting mechanism. The instant retrigger unit circumvents this effect by forcing additional count signals whenever the digital pulse exceeds a preset retrigger interval [5,6]. Both detectors mentioned above incorporate charge-sensitive front-end amplifiers, which are not designed and optimized for ultra-low-energy detection. To obtain shorter response pulses, these amplifiers trade charge gain for reduced pulse widths, which leads to relatively large equivalent noise charge (ENC). Consequently, these detectors typically achieve minimum energy resolutions ranging from 1.37 keV to 2.70 keV [3,4,5,6]. As SRSs expand to cover a broader energy range, the demand for readout chips with lower-energy resolution increases.

To address this challenge, a pixel readout ASIC (application-specific integrated circuit) with a small pixel size and low ENC noise is proposed, aimed at extending the operating range of hybrid SPC detectors to lower energies. A small-scale prototype chip integrating a 2 × 16 pixel matrix with a 50 μm pixel pitch was designed to validate the readout architecture and pixel performance. In Section 2, the architecture and readout scheme of the prototype chip are presented. Section 3 describes in detail the pixel designs implemented in the matrix. Section 4 presents the measurement results. Section 5 summarizes this work.

## 2. Chip Overview

**Figure 1:**
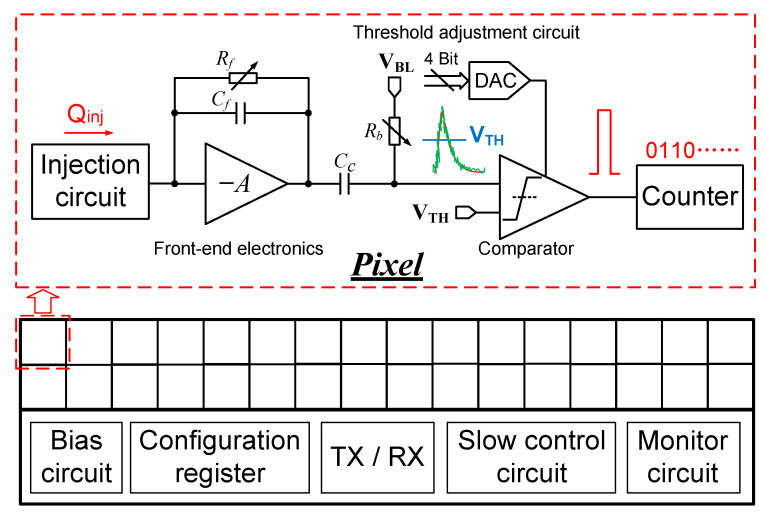
Architecture of the prototype chip operating in SPC mode.

The prototype chip architecture operating in the SPC mode is shown in Figure 1. The chip consists of two principal blocks: a 2×16 pixel matrix and the supporting peripheral circuitry. Each pixel integrates a front-end amplifier for detecting the input charge, a comparator with a trimming DAC for threshold discrimination, and a pair of 15-bit pixel counters that can be alternately configured between counting and readout modes to enable continuous readout. A charge injection circuitry is embedded in each pixel to emulate signal charges for electronic testing and detector calibration. The design details of the pixel matrix are presented in Section 3. Upon injection of a charge signal, the front-end amplifier produces a voltage pulse. The pulse is then coupled to the comparator input node through a capacitor $C_{c}$. If the pulse amplitude exceeds the preset threshold $V_{TH}$, the comparator generates a digital pulse. The rising edge of this digital pulse increments the pixel counter, which retains the count until readout.

**Figure 2:**
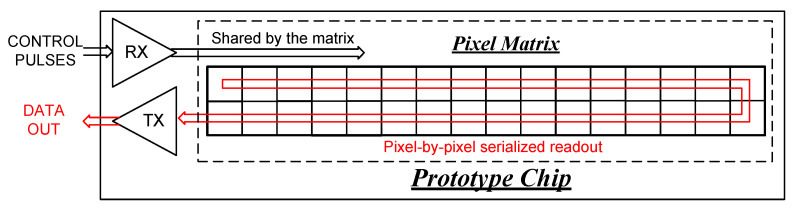
Functional control and data readout scheme of the pixel matrix.

Figure 2 shows the control and data readout schemes for the pixel matrix. Functional control is effected by a common set of pulses received from multiple digital receivers, and the resulting digital bits of each pixel are serialized and transmitted off-chip through a digital output driver. The Analog biasing for the pixel circuitry is supplied by seven 10-bit current digital-to-analog converters (DACs) located at the chip periphery. Slow control is implemented via a Serial Peripheral Interface (SPI) that configures both peripheral circuitry and individual pixels. Analog signals from critical nodes on the chip are accessible for monitoring during testing and debugging.

**Figure 3:**
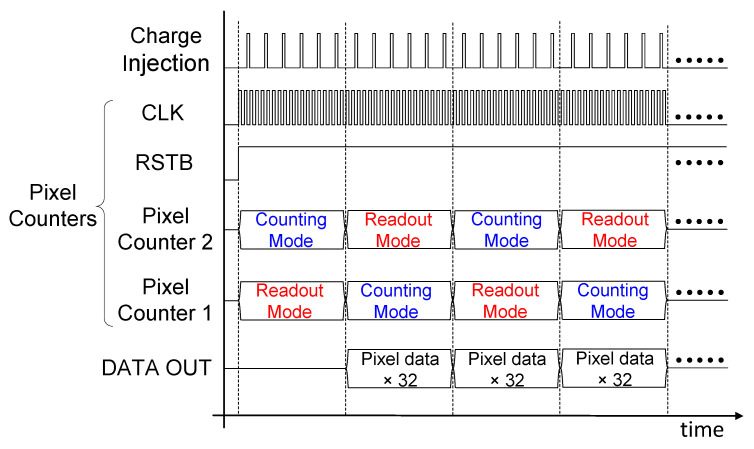
Timing diagram for the small-scale prototype chip.

Figure 3 shows the timing diagram of the prototype chip. As described above, each pixel is equipped with a pair of 15-bit pixel counters that alternate between counting and readout modes to enable continuous readout. In counting mode, the incoming charge signals are discriminated and then accumulated. In readout mode, the count data from the previous period for all 32 pixels (15-bits data per pixel) are serialized and shifted sequentially through the DATA OUT port. RSTB is the forced-reset signal (low active), and CLK determines the readout rate.

**Figure 4:**
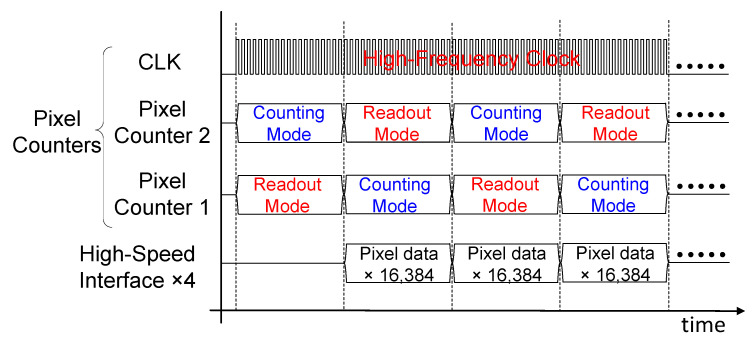
Timing diagram for expected full-scale chip.

In the anticipated full-scale chip, which will include a 256×256 pixel matrix, data buffers and high-speed serial link circuitry will be added in the periphery to enable similar operation (See Figure 4). Driven by a high-frequency clock, each 64×256 sub-matrix will share a high-speed serial interface to readout 16,384 pixels (15-bits data per pixel).

## 3. Pixel Matrix

This section presents the details of the pixel design: Section 3.1 describes the front-end amplifier; Section 3.2 the charge-injection circuit; Section 3.3 the comparator with a threshold-adjustment DAC; Section 3.4 the pixel counter, which toggled between counting and readout modes; and Section 3.5 the pixel layout.

### 3.1. Front-End Amplifier

**Figure 5:**
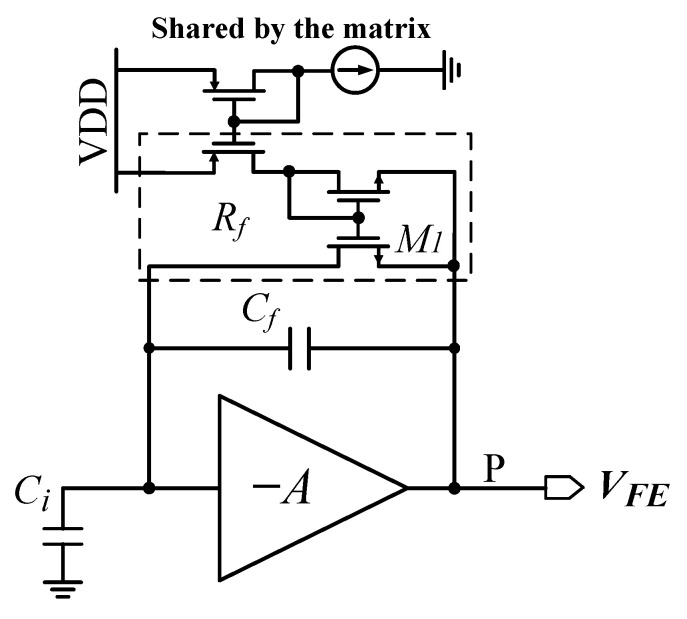
Schematic of the pixel front-end amplifier.

Figure 5 shows the schematic of the front-end amplifier, which is designed for electron collection. The main amplifier is a gain-boosting-based design, similar to the one used in [7]. The front-end employs a current-mirror arrangement to bias transistor M1 with a relatively small current in the range of 0.1–10 nA (nominal bias 1 nA), thus achieving a discharge current source with a tunable effective resistor $R_{f}$.

During detection, the X-ray pulse converts its energy into charge signals $Q_{in}$ within the sensor material, which are integrated into the feedback capacitor $C_{f}$ of the front-end amplifier. At the same time, the accumulated charge in $C_{f}$ is discharged through the effective resistor $R_{f}$, thus producing a voltage pulse. According to [8,9], the pulse response in terms of input charge signals $Q_{in}$ can be expressed as:

(1) $$V_{out}=\frac{1}{C_{f}}\cdot \frac{\tau_{F}}{\left(\tau_{R}-\tau_{F}\right)}\cdot \left(e^{-\frac{t}{\tau_{R}}}-e^{-\frac{t}{\tau_{F}}}\right)\cdot Q_{in},$$

where $\tau_{F}$ is the feedback time constant of the front-end amplifier

(2) $$\tau_{F}=R_{f}\cdot C_{f},$$

and $\tau_{R}$ is the pulse rise time constant of the front-end amplifier

(3) $$\tau_{R}=\frac{C_{i}C_{l}+C_{i}C_{f}+C_{l}C_{f}}{g_{m1}\cdot C_{f}}\approx \frac{C_{i}C_{l}}{g_{m1}\cdot C_{f}}.$$

where $C_{i}$ is the total capacitance at the front-end amplifier input node, including the sensor capacitance; $C_{l}$ is the total load capacitance at node P; and $g_{m1}$ is the transconductance of the main amplifier. According to Equation (1), the front-end charge gain $G_{q}$ and ENC noise can be calculated as:

(4) $$G_{q}=\frac{V_{out}}{Q_{in}},$$

(5) $$ENC=\frac{1}{G_{q}}\cdot V_{FErms}.$$

where $V_{FErms}$ is an rms noise voltage at the front-end amplifier output. From the circuit analysis mentioned above, the ENC noise and pulse width of the front-end amplifier depend on $C_{f}$, $\tau_{R}$, and $\tau_{F}$. A higher charge gain leads to wider output pulses; therefore, the front-end design must compromise between low ENC noise and short pulse widths.

The detector count rate is characterized by the photon events recorded per pixel size per second when the hybrid SPC detector lost 10% input rate parameter. This value is limited by the pulse width of the front-end amplifier: shorter pulses reduce per-event processing time, enabling pixels to process charge signals more rapidly. The front-end amplifier presented in this work is specifically optimized for low-energy detection. Feedback capacitance $C_{f}$ (1.2 fF) and transistor M1 were carefully selected to provide a relatively large charge gain $G_{q}$, accepting wider pulse widths as a trade-off to achieve excellent ENC noise and lower-energy resolution.

**Figure 6:**
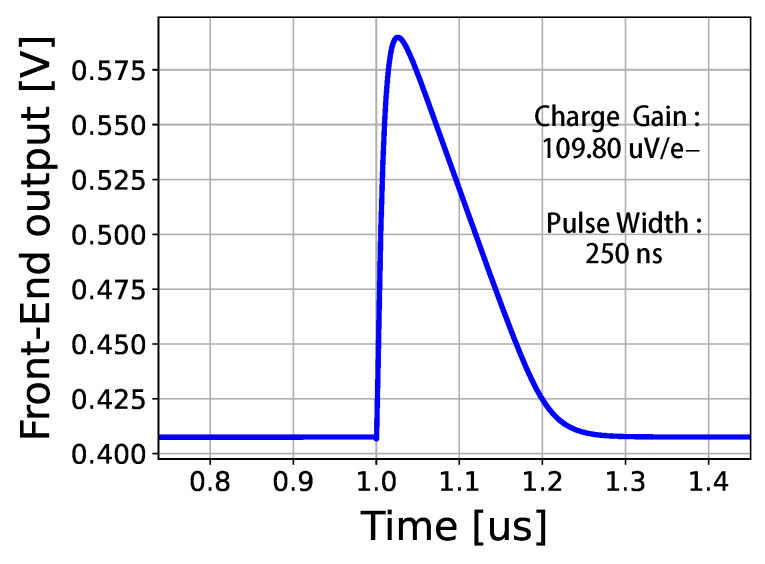
Front-end amplifier output waveform from the pixel post-layout simulation.

**Figure 7:**
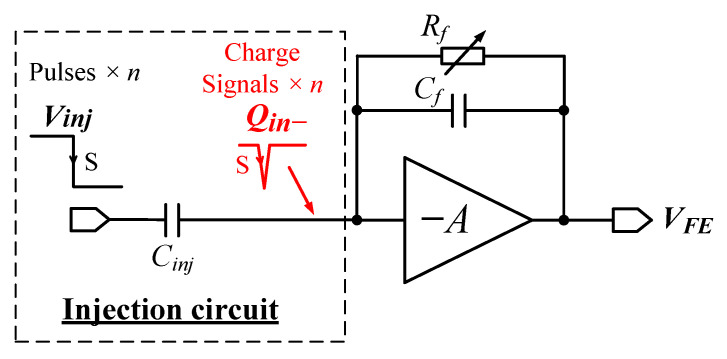
Illustration of the injection circuit.

Figure 6 shows the front-end amplifier output waveform from the pixel post-layout simulation. For simulation, the input charge was provided by the injection circuit (see Figure 7) and corresponds to a charge of approximately 1656 e^−^. The results indicate a pulse width of 250 ns and a charge gain of 109.80 μV/e^−^. The pixel design presented in this work follows a paralyzable dead-time model [10], and its theoretical output count rate $N_{out}$ can be expressed as:

(6) $$N_{out}=N_{in}\cdot e^{-N_{in}\cdot \tau_{p}},$$

where $N_{in}$ is the incident photon rate, and $\tau_{p}$ is the dead time, which is approximately equal to the pulse width of the front-end. Therefore, the theoretical maximum count rate for this work can be estimated:

(7) $$N_{max}\left[\frac{M\mathrm{cps}}{{\mathrm{mm}}^{2}}\right]=\frac{1}{e\cdot \tau_{p}}\cdot \frac{1}{pixel\;area}.$$

where $N_{max}$ is the theoretical maximum count rate of this work, which can be calculated as 588.6 Mcps/mm^2^. Compared with the reported maximum count rates of Medipix 3 (826 Mcps/mm^2^) and Eiger (747 Mcps/mm^2^) [11], the estimated maximum count rate in this work is worse.

### 3.2. Charge Injection Circuit

The charge injection circuitry is necessary to evaluate circuit performance when the sensor is not connected and is also essential for detector calibration after the readout chip has been assembled with the sensor. As illustrated in Figure 7, a charge signal injection method is implemented within each pixel. This voltage method applies an adjustable voltage step through a capacitor $C_{inj}$ to the pixel input node, generating a total charge $Q_{in}$ given by:

(8) $$Q_{in}=V_{inj}\cdot C_{inj}.$$

where $V_{inj}$ is the amplitude of the voltage step. Assuming a mean ionizing energy of 3.62 eV for silicon [12], the conversion factor in the silicon sensor is approximately 0.276 e^−^/eV. With $C_{inj}$ valued at approximately 9.3 fF in the pixel design, injection circuitry can generate test pulses equivalent to different X-ray photon energies for chip measurement and detector calibration.

### 3.3. Comparator with Threshold-Adjustment DAC

**Figure 8:**
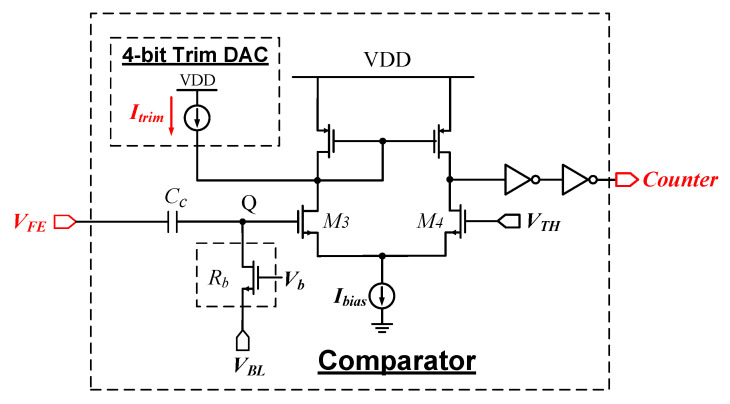
Schematic of the comparator with a threshold-adjustment DAC.

Figure 8 shows the schematic of comparator design with a threshold-adjustment DAC. An MOS resistor $R_{b}$ is used at node Q; its effective resistance of $R_{b}$ is controlled by the bias voltage $V_{b}$. The coupling capacitor $C_{c}$ isolates the DC voltage set by the front-end amplifier; the DC baseline at node Q is therefore established by the external global voltage $V_{BL}$ (common to all pixels) and can be fine-tuned through the resistor $R_{b}$. The external voltage $V_{TH}$ sets the global threshold for the pixel matrix.

The Trim DAC injects an adjustable current $I_{trim}$ into the drain of the input transistor M3, redistributing the bias current $I_{bias}$ between M3 and M4. Thus, the decision threshold of the comparator is shifted. Each Trim DAC can be configured independently through a 4-bit register embedded in the pixel, allowing per-pixel correction of process-induced offsets to eliminate the fixed-pattern noise across the matrix.

### 3.4. Pixel Counter

**Figure 9:**
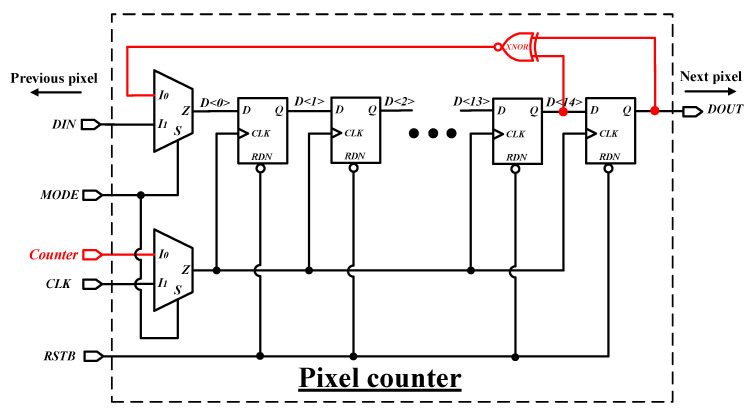
Schematic of the pixel counter with readout mode and counting mode.

Figure 9 presents the schematic of the pixel counter, which can be toggled between readout mode and counting mode through simple logic. The design consists of 15 registers, one XNOR gate, and two multiplexers. When the MODE signal is low, the counter operates in counting mode (indicated in red): the circuitry implements a 15-bit LFSR (linear feedback shift register) counter through the XNOR gate. The pulses from the comparator increment the LFSR, causing the counter to record the count values in a pseudo-random sequence. When the MODE signal is high, the counter switches to readout mode: each pixel in the matrix is inserted into a long serial chain of registers (See Figure 2), and the stored counts are shifted off the chip under CLK control. RSTB is the forced-reset signal. Each pixel integrates 2 counters that alternate between the counting and readout modes, ensuring continuous readout.

### 3.5. Pixel Layout

**Figure 10:**
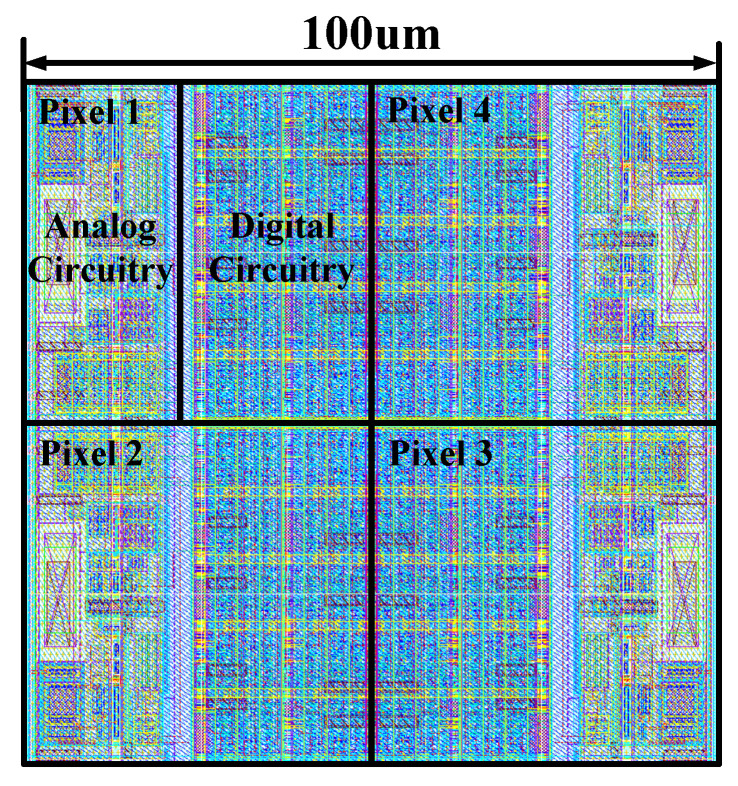
Layout of 2 by 2 pixels for the prototype chip.

Figure 10 shows the layout of 2 × 2 pixels for the prototype chip. Each pixel occupies approximately 50 μm × 50 μm, and the functional circuitry of each pixel is annotated on the layout. To minimize interference, the layout segregates the analog and digital domains: digital blocks are grouped at the center of the double column, while analog blocks are placed on both sides.

## 4. Measurement Results

**Figure 11:**
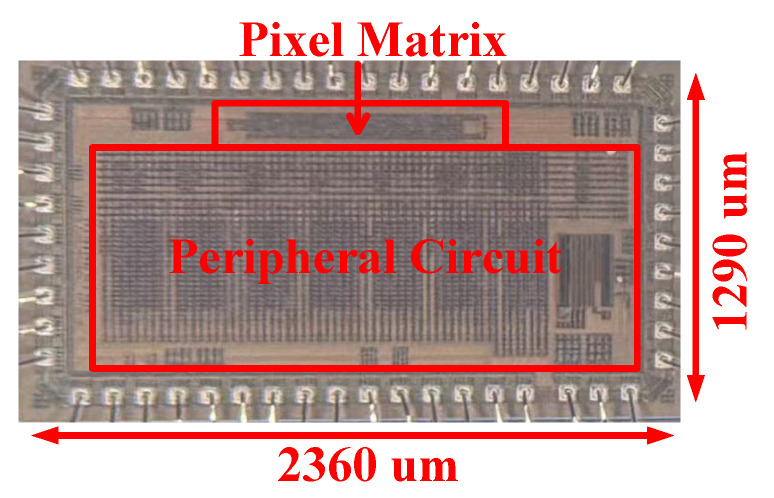
Micrograph of the prototype chip.

**Figure 12:**
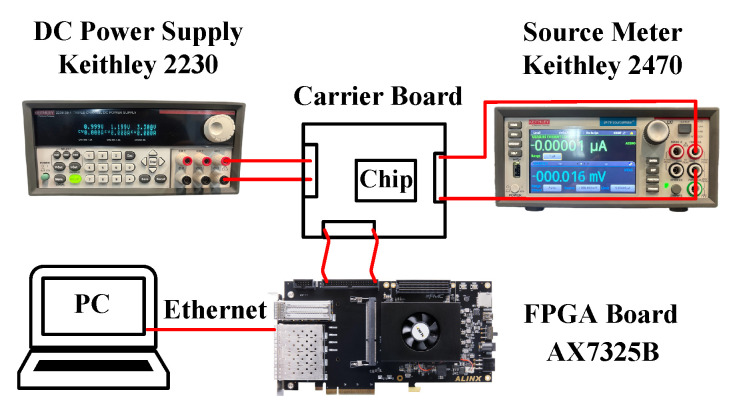
The chip measurement setup.

The prototype chip was designed and manufactured in a 130 nm CMOS process. Figure 11 shows the chip photograph, and Figure 12 illustrates the measurement setup. This chip is wire-bonded to a carrier board that supplies the required reference voltages and currents. A multi-channel DC power supply provides 3.3 V and 1.2 V to the I/O and core circuits of the chip, respectively. An adjustable voltage from a source meter drives the pixel injection circuit. An AX7325B FPGA development board from ALINX (Shanghai, China) [13] is used to configure the chip and acquire the measurement data. Section 4.1 describes the measurement methodology and calibration for the pixel matrix; Section 4.2 presents the gain measurement results; and Section 4.3 reports the noise measurement results.

### 4.1. Measurement Methodology and Correction

The electronic response of each pixel was characterized using the voltage injection method described in Section 3.2. For the measurements, the global baseline voltage $V_{BL}$ was set to 0.7 V, and the electrical stimulus was set equal to the charge produced by absorption of a 6 keV photon within the silicon sensor (approximately 1656 e^−^). Pixel performance was evaluated using multiple threshold scans: for each scan, a fixed comparator threshold voltage was chosen, and the continuous charge signals were injected into a single pixel repeatedly while its responses were counted and readout. Sweeping the threshold across a range and plotting the recorded counts versus threshold contains information about pixel performance.

**Figure 13:**
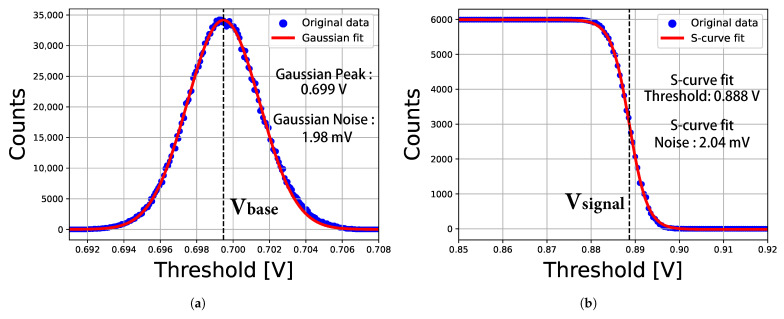
Exemplary threshold scans for a single pixel (pixel number 3) with (**a**) no input charge applied. (**b**) continuous input charge signals of a given photon energy (6 keV).

Figure 13 shows the threshold scans for an example pixel (pixel number 3), obtained both without input charge and with continuous input charge signals of a given photon energy (6 keV). The baseline distribution was fitted with a Gaussian function; the mean (Gaussian Peak $V_{base}$) and standard deviation $\sigma_{b}$ (Gaussian Noise) of the fit are indicated in Figure 13a. And the integral spectrum (so-called “S-curve”) of the signal-peak distribution was fitted to the error function [8]; the mean (S-curve fit Threshold $V_{signal}$) and the standard deviation $\sigma_{s}$ (S-curve fit Noise) of the fit are indicated in Figure 13b. The standard deviation $\sigma_{b}$ of the baseline distribution defines the pixel output noise, and the noise can also be deduced from the S-curve fit $\sigma_{s}$. These two noise-estimation methods yield nearly identical results. The pixel response amplitude is defined as the separation between the comparator threshold $V_{signal}$ for a 6 keV photon and the Gaussian-fitted baseline voltage $V_{base}$. The charge gain $G_{q}$ of the selected pixel can be calculated as:

(9) $$G_{q}=\frac{V_{signal}-V_{base}}{Q_{in}}$$

where $Q_{in}$ is the input charge per injection, approximately 1656 e^−^.

**Figure 14:**
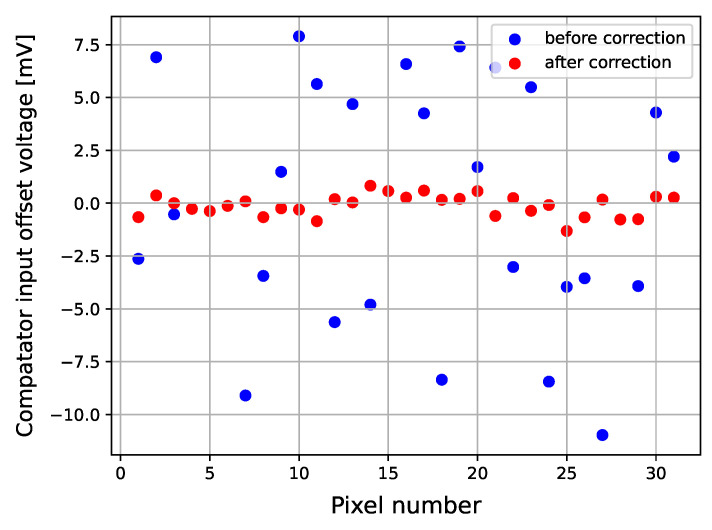
Comparator DC offset voltage versus pixel number before and after correction.

Figure 14 shows the measured DC offset relative to the global baseline voltage $V_{BL}$ before and after correction. The mean value of the baseline distribution from a Gaussian fit (Gaussian Peak $V_{base}$) defines the comparator DC offset (see Figure 13a). Before calibration, the comparator offsets across the pixel matrix exhibited a standard deviation of 5.24 mV. After configuring the Trim DACs per pixel, the standard deviation was reduced to 0.50 mV, effectively eliminating the fixed pattern noise of the matrix. The comparator offset correction step is particularly beneficial, where a single global threshold is applied to all pixels.

### 4.2. Gain

**Figure 15:**
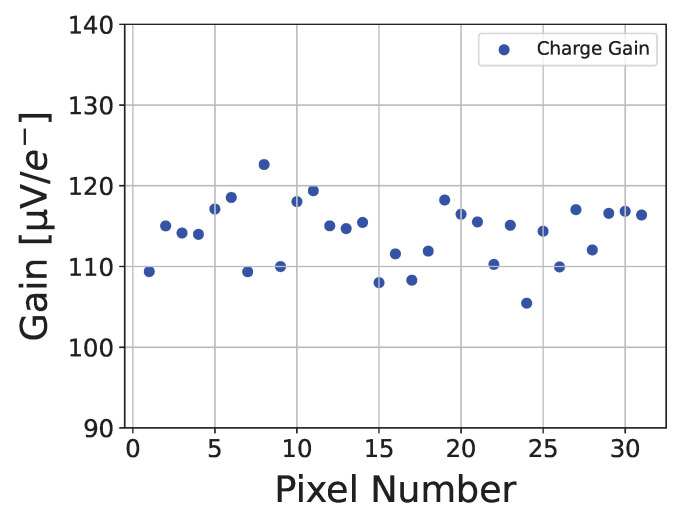
The measured charge gain $G_{q}$ versus pixel number.

The measurement method described in Section 4.1 will be applied to evaluate the charge gain $G_{q}$ of the entire pixel matrix. Figure 15 presents the charge gain distribution across the matrix. The measurement results show a relatively uniform charge gain $G_{q}$ within the matrix: all pixel values range from 105 μV/e^−^ to 125 μV/e^−^, with a mean of 114.09 μV/e^−^ and a standard deviation of 3.84 μV/e^−^.

### 4.3. Noise

**Figure 16:**
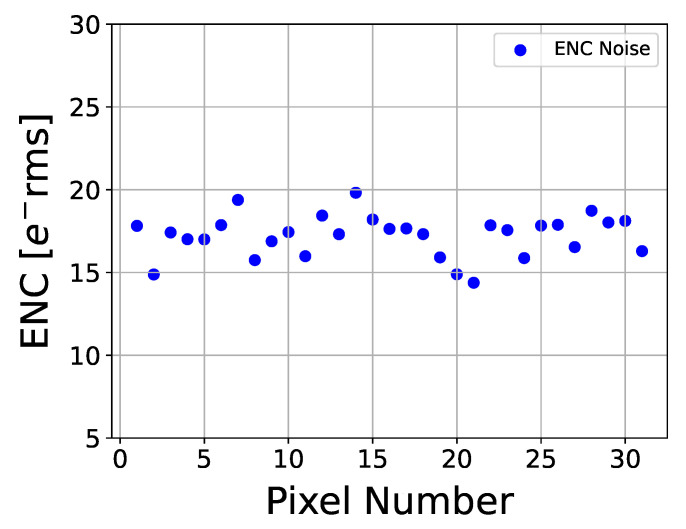
The measured ENC noise versus pixel number.

Using the measurement methods described in Section 4.1, the output noise of each pixel can be obtained. The charge gain $G_{q}$ of each pixel is provided in Figure 15. The measured ENC noise, defined as the ratio of output noise to charge gain, was calculated for all pixels. Figure 16 shows the measured noise ENC distribution across the pixel matrix. The results indicate good uniformity: ENC noise values range from 14 e^−^rms to 20 e^−^rms, with a mean of 17.21 e^−^rms and a standard deviation of 1.24 e^−^rms.

Note that the ENC noise measurements were performed on the prototype chip without the bump-bonded sensor. In the expected full-size readout chip with the bump-bonded sensor, the coupling capacitance of the sensor chip will reduce the charge gain $G_{q}$, and consequently lead to worse ENC noise. Assuming a silicon sensor thickness of 300 μm and a coupling capacitance of 50 fF, a rough estimate of expected performance can be obtained by combining sensor modeling with pixel post-layout simulation. The results indicate that the pixel charge gain $G_{q}$ is reduced by 10.62%, leading to an increase of 10.62% ENC noise.

To ensure good energy resolution, the chip ENC noise should remain well below the charge generated within the silicon detector when a single photon is absorbed. A 1 keV photon absorbed in the silicon sensor generates a charge signal approximately 276 e^−^, which substantially exceeds the measured ENC noise. Therefore, the prototype chip has the potential to achieve the lower-energy resolution required for synchrotron hybrid SPC detectors.

### 4.4. Comparison of SPC Readout Chips

Table 1 summarizes a comparison of the SPC readout chips in sub-micron CMOS technology. Compared with existing SPC readout chips, the main advantages of the presented chip are its smaller pixel size and lower ENC noise. However, its principal drawbacks are relatively high per pixel power consumption and a fixed bit depth. The Medipix-series and Eiger-series support selectable bit depth of up to 24 and 32 bits, respectively; configurable bit depth will be incorporated in the expected full-size chip of this work.

## 5. Conclusions

This paper presents the design and characterization of a prototype pixel readout ASIC with a small pixel size and low noise, developed for synchrotron hybrid SPC detectors. The chip was manufactured in a 130 nm CMOS process and integrates a small-scale pixel matrix with 50 μm × 50 μm pixel size. Each pixel consists of a front-end amplifier, a discriminator, a charge injection circuitry, and a pair of 15-bit counters that can be configured alternately between the counting mode and readout mode to enable continuous readout. Measurements were performed on the prototype chip without the bump-bonded sensor. The results demonstrate relatively uniform ENC noise across the matrix, with all pixel values ranging from 14 e^−^rms to 20 e^−^rms. The overall ASIC performance, compared with other designs, is summarized in Table 1. The main advantages of the presented chip are its smaller pixel size and lower ENC noise, indicating that the pixel design has the potential to deliver a lower-energy resolution for synchrotron hybrid SPC detectors.

## Figures and Tables

**Table 1:** Comparison of the single photon-counting chips in sub-micron CMOS technology.

Chip	Medipix 3RX	PXD23K	Eiger	Eiger2	This Work
[Ref]	[3,11]	[11,14]	[5,15]	[6]	
Process	130 nm	130 nm	250 nm	-	130 nm
Pixel Size [μm^2^]	55×55	75×75	75×75	75×75	50×50
Bit Depth	24-bit (max)	14-bit	12-bit (max)	32-bit (max)	15-bit
Power/pix. [μW]	9	25	10	-	12.2 **
Frame Rate [kHz]	-	-	24 (4 bit mode)	98 (ROI mode)	68 (32 pixels)
ENC [e^−^rms]	80	89 ^#^	110	-	20 ^#^
Energy Resolution *	1.37 keV	-	2.1 keV	2.7 keV	-

* The minimum single photon energy the hybrid detector can discriminate. ** Simulation results. With only 32 pixels, peripheral power consumption dominates the measured supply current. ^#^ Measured without bump-bonded sensor.

## Data Availability

The original contributions presented in this study are included in the article. Further inquiries can be directed to the corresponding author.
